# Disproportionately low authorship by women urologists in sexual medicine journals

**DOI:** 10.1093/sexmed/qfaf095

**Published:** 2025-12-01

**Authors:** Sonam Saxena, Akhil Peta, Michael Raver, Simon Gelman, Matthew Yang, Maie Abdel-Wahab, Nolan Holbrook, Sophia Zhang, Jamie Chen, Sarah Brink, David Shin

**Affiliations:** Department of Urology, Hackensack University Medical Center, Hackensack, NJ 07662, United States; Department of Urology, Hackensack University Medical Center, Hackensack, NJ 07662, United States; Department of Urology, Hackensack University Medical Center, Hackensack, NJ 07662, United States; Department of Urology, Hackensack University Medical Center, Hackensack, NJ 07662, United States; Department of Urology, Hackensack Meridian School of Medicine, Nutley, NJ 07110, United States; Department of Urology, Hackensack Meridian School of Medicine, Nutley, NJ 07110, United States; Department of Urology, Hackensack Meridian School of Medicine, Nutley, NJ 07110, United States; Department of Urology, Hackensack Meridian School of Medicine, Nutley, NJ 07110, United States; Department of Urology, Hackensack Meridian School of Medicine, Nutley, NJ 07110, United States; Department of Urology, Hackensack University Medical Center, Hackensack, NJ 07662, United States; Department of Urology, Hackensack University Medical Center, Hackensack, NJ 07662, United States

**Keywords:** sexual medicine, gender disparity, women authorship

## Abstract

**Background:**

The recent efforts to increase the proportion of women in urology have demonstrated success; it is equally important to evaluate success of these efforts in urologic sub-specialized fields as well.

**Aim:**

This study aims to evaluate the impact of these efforts and the representation of women urologists in the sexual medicine literature.

**Methods:**

Original research manuscripts from three prominent sexual medicine journals from 2013 to 2023 were collected. Manuscripts submitted under Paraphilia subsections, as well as systematic reviews, meta-analyses, communications, commentaries, replies, and those involving psychometrics were excluded. Proportion of women authors in these journals’ literature was determined by searching names of first and last authors on the Internet.

**Outcomes:**

This study evaluates whether authorship by women in sexual medicine literature has increased following recent efforts to enhance their representation in urology and related fields.

**Results:**

1065 of 2701 (39%) collected manuscripts meeting criteria were from first (FA) or last authors (LA). Women were significantly less likely to be FA or LAs across these manuscripts (*P*< 0.001).

Further, variables such as author degree, field, profession, and institution type all demonstrated significant gender disparities in FA and LA as well. Representation of women FAs and LAs was lowest in urology (15%, 5%) compared to obstetrics and gynecology (64%, 55%) and psychology (68%, 63%).

**Clinical Implications:**

These disparities in sexual medicine publications have important consequences for women urologists; these opportunities often impact future career advancement in academia.

**Strengths and Limitations:**

Gender identification relied on traditional markers (such as names and pronouns), which may not fully capture the diversity of gender identities, we sought to mediate that by using a binary identification method. The analysis was also limited to first and last authorship, which may not capture contributions from middle authors.

**Conclusion:**

Although the push to increase the proportion of women urologists has shown success, there are still significant disparities seen in publication of sexual medicine literature. Potential avenues for improvement include mentorship and targeted institutional support.

## Introduction

Gender equity in academic medicine is critical not only for ensuring fair opportunities but also for fostering diverse perspectives. However, certain specialties, such as urology, suffer from a large disparity. Women are historically underrepresented in urology.^1^ A recent (2023) report shows that only 11.8% of urology attendings are women, a 4.1% increase since 2014, which showed a proportion of 7.7%.^2^^,^^3^ However, the trend is slowly changing, with women representing 37% of the accepted residency class of 2028^4^ and 45% of the accepted class of 2029.^5^

As a result of this imbalance in the workforce, it is obvious that women are also underrepresented in urology authorship. This disparity is especially highlighted when comparing genders of last authors of manuscripts, positions typically denoting seniority. Arbelaez *et al*. recently showed that only 15% of last authors in urology literature from 2015 to 2020 were women. Further, women were significantly less published in high-impact urology journals.^6^ This disparity can have significant downstream consequences, given that publications are often a key metric for obtaining faculty and leadership positions.^7^

This trend extends to the urologic subspecialty of sexual medicine, where manuscript authorship also demonstrates a significant gender discrepancy. For example, a study published in *Journal of Sexual Medicine* (*JSM*) (2002-2020) showed that women represented 30.7% of first authors and 21.3% of last authors in sexual medicine journals. These numbers suggest active participation in sexual medicine research, although their representation decreases in more senior authorship positions.^8^

Given the increased numbers of women joining the field of urology, it is important to ensure that they are supported academically across all subspecialties. This study examines the following: Have the initiatives to increase the numbers of women in urology translated to an increase in women authors represented in the sexual medicine literature?

## Methods

Original research manuscripts in *JSM*, *Sexual Medicine* (*SM*), and *International Journal of Impotence Research* (*IJIR*) from 2013 to 2023 were reviewed, original research manuscripts were included. First author (FA) and last author (LA) characteristics were collected. Manuscripts with single authors were classified under first author for the analysis. Manuscripts submitted under Paraphilia subsections, as well as systematic reviews, meta-analyses, communications, commentaries, replies, and those involving psychometrics were excluded.

An Internet search was performed to best determine author gender by listed pronouns, picture, or traditionally gendered name. We recognize this method of determining gender based on conventional gender presentation is imperfect and may not represent the full spectrum of gender diversity.

Fisher’s exact tests and Chi-square analyses were performed using SPSS (IBM Corporation, 2024, Version 30.0) to assess association between gender and studied characteristics.

IRB approval was not required for this study, given that there was no patient-specific information utilized.

## Results

Author characteristics are presented in Table 1.

**Table 1:** Characteristics of first and last authors across sexual medicine journals in North America from 2013 to 2023.

**Women authorship in sexual medicine journals—North America, 2013-2023**						
	**First author**			**Last author**		
	**Woman *n* = 398** **[*n* (%)]**	**Man *n* = 595** **[*n* (%)]**	***P*-value**	**Woman *n* = 286** **[*n* (%)]**	**Man *n* = 730** **[*n* (%)]**	***P*-value**
**Journal**			<.001^a^			<.001^a^
*JSM*	312 (45)	387 (55)		230 (32)	484 (68)	
*SM*	51 (39)	81 (61)		39 (29)	96 (71)	
*IJIR*	35 (22)	127 (78)		17 (10)	150 (90)	
**Year**			.42^a^			.22^a^
2013	44 (39)	68 (61)		27 (23)	91 (77)	
2014	34 (38)	55 (62)		27 (29)	65 (71)	
2015	44 (47)	50 (53)		30 (32)	63 (68)	
2016	30 (45)	36 (55)		22 (35)	40 (65)	
2017	26 (39)	40 (61)		24 (34)	46 (66)	
2018	33 (37)	57 (63)		15 (16)	79 (84)	
2019	31 (33)	64 (67)		30 (30)	70 (70)	
2020	50 (44)	64 (56)		37 (31)	83 (69)	
2021	34 (35)	63 (65)		26 (27)	72 (73)	
2022	38 (37)	64 (63)		30 (29)	74 (71)	
2023	34 (50)	34 (50)		18 (28)	47 (72)	
**Author degree**			<.001^b^		5 (36)	<.001^b^
Bachelor’s	42 (40)	62 (60)		9 (64)	3 (43)	
Master’s	38 (76)	12 (24)		4 (57)	138 (46)	
PhD	153 (63)	89 (37)		162 (54)	566 (86)	
MD/equivalent	129 (24)	411 (76)		93 (14)	13 (48)	
Other	30 (70)	13 (30)		14 (52)		
**Author field**			<.001^c^			<.001^c^
Urology	60 (15)	329 (85)		25 (5)	461 (95)	
OB-GYN	35 (64)	20 (36)		38 (55)	31 (45)	
Psychology	98 (68)	47 (32)		87 (63)	51 (37)	
Other	44 (54)	37 (46)		62 (64)	35 (36)	
**Author profession**			<.001^c^			<.001^b^
Urology attending	14 (9)	143 (91)		25 (5)	455 (95)	
Urology fellow	17 (23)	58 (77)		1 (33)	2 (67)	
Urology resident	29 (19)	126 (81)		2 (100)	0 (0)	
Non-urology attending	45 (49)	46 (51)		63 (43)	84 (57)	
Non-urology fellow	6 (100)	0 (0)		1 (100)	0 (0)	
Non-urology resident	9 (47)	10 (53)		-	-	
Medical student	25 (28)	65 (72)		2 (50)	2 (50)	
Non-physician/researcher	231 (68)	109 (32)		165 (54)	140 (46)	
**Author institution**			<.001^a^			<.001^a^
Public	228 (51)	215 (49)		170 (39)	266 (61)	
Private	147 (30)	347 (70)		89 (18)	405 (82)	

a Pearson’s Chi-squared test.

b Fisher’s exact test for count data with simulated *P*-value (based on 2000 replicates).

c Fisher’s exact test.

**Figure 1:**
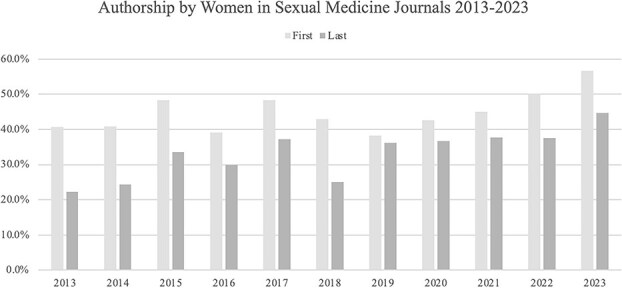
Trends of proportions of women authors in sexual medicine journals from 2013 to 2023.

Trends in women authorship as FA or LA from 2013 to 2023 are shown in Figure 1. One thousand sixty-five of 2701 (39%) manuscripts meeting criteria were from first (FA) or last authors (LA). Overall, women were significantly less likely to be FAs or LAs (*P* < 0.001); *JSM* showed the greatest proportion of both women FAs and LAs (45%, 32%), followed by *SM* (39%, 29%), then *IJIR* (22%, 10%). Significant gender disparities were seen in author degree, field, profession, and institution (all *P* < .001). There were fewer women FAs and LAs among medical doctors (24%, 14%) compared to PhDs (63%, 54%).

Representation of women FAs and LAs was lowest in urology (15%, 5%) compared to obstetrics and gynecology (OB-GYN) (64%, 55%) and psychology (68%, 63%). Despite comprising >50% of medical school cohorts, only 28% of medical student FAs were women. In public institutions, FA gender was nearly evenly split (51:49) and women comprised 39% of LAs; however, women were only 30% of FAs and 18% of LAs in private institutions. The analysis of gender correlation shows women FAs are more positively associated with women LAs and vice versa (OR 4.82, CI, 3.57-6.54, *P* < .001).

## Discussion

In recent years, the field of urology has made significant progress in promoting gender equity through initiatives such as mentorship programs, dedicated research grants, and policy changes designed to encourage women to join the urology workforce. Representation of women in urology has steadily increased over the past few decades from 7.2% to 26%. With this increase in representation, there has been an encouraging increase in the proportion of senior authors who are women.^6^^,^^7^

However, this progress has not translated into leadership roles in academic publications, which is an important factor in career advancement. One possible explanation for the slow progress is that the field of urology is still within its early stages of becoming a diverse and representative workforce. Among urologists <45 years old, 22% are women compared to just the 1% of urologists 65 and older^2^; it is possible that many women who have entered the field are still in the early stages of their career and have not yet gathered enough experience to obtain those editorial roles. Efforts to increase this representation even for younger urologists should optimize mentorship opportunities, which can help early-career urologists navigate the academic publishing landscape, gain experience in peer review, and build networks.

Disparities within urology persist across its subspecialties, including sexual medicine. This study found that within the sexual medicine literature, urology demonstrated the lowest representation of FAs and LAs (15%, 5%) who are women compared to other specialties such as OB-GYN (64%, 55%) and fields like psychology (68%, 63%); one hypothesis posits that this is likely due to higher proportions of women in OB-GYN and psychology. For example, in 2023, women earned 74.7% of Psychology doctorates awarded by US universities.^9^ Additionally, in academic OB-GYN, women represented 57% of attending physicians as of 2018^10^; these two fields with higher representation of women were found to also exhibit stronger authorship by women in our study. As women leadership in urology grows, we hope to see a similar increase in women authorship.

This study collected manuscripts from *JSM*, *SM*, and *IJIR* from 2013 to 2023 and found that the proportion of FA that were women increased (39% to 50%). However, our finding of persistent underrepresentation of LAs who are women (2013: 23%, 2023: 28%) highlights the slow pace of change in senior academic roles.

Last authorship, often indicative of seniority and leadership, is crucial for career advancement and recognition. There is a known disparity between sexes when it comes to leadership positions in academia. A 2019 study revealed that, up to that point, no major urology journal had ever had a female editor-in-chief.^11^ A cross-sectional observational study of US Urology residency programs conducted in 2016-2017 also showed that only 3.3% of department chair positions were held by women.^12^ As of 2019, only 4.5% of urology department chairs or chiefs were women,^13^ indicating a slowly increasing trend of women in these leadership roles.

When taking degree of education into account, this study found that women who were medical doctors (MDs) or equivalents were the least likely to have LA positions compared to MDs or equivalents who were men (women: 14% vs. men: 86%). This was different from the other stages of career examined; at the bachelor’s, master’s, and PhD levels, this study found a higher percentage of women authors than men authors. This signals that the medical community is severely lacking in increasing authorship of sexual medicine literature by women.

To build on this, this study also analyzed studies by author profession or stage of career. Similarly, we found that urology and non-urology attending LAs were more often men (urology: 95%, non-urology: 57%), as was the same for fellows (urology: 67%, non-urology: 57%). This further emphasizes the need for improvement in equal representation in the urology field.

Lastly, we examined whether the type of institution differed between authorship and gender. We found that public institutions had a higher percentage of women FA compared to private institutions (51% vs. 30%), which may be attributed to a higher percentage of women LA seen in public (39%) versus private (18%) institutions. This further highlights the power of mentorship creating academic opportunities for women.

### Future action

Reducing the gender disparity in authorship will require a multifaceted approach. Current literature suggests that the use of a double-blinded review process significantly increases authorship by women (*P* < .001).^6^ Institutional support, including mentorship programs, faculty development programs, and bias training for review committees, have also been offered as ways to increase authorship by women.^14^ Mentorship and faculty development programs can benefit authorship by providing advice in grant writing and leadership as well as encouraging perseverance.^15^

Our study also provides another avenue for increasing women’s authorship by showing that gender concordant mentorship provides a higher likelihood of seeing women FAs co-authoring with women LAs. This trend was corroborated in multiple other studies and specialties: both pediatrics and OB-GYN literature—notably two fields with a predominance of women—have described the impact of women’s co-authorship significantly increasing first and last authorship by women.^16–18^ Further, this trend worked both ways in our study, as women LAs were more likely to be associated with women FAs (*P* < .001).

Future research on this topic will need to focus on understanding the barriers contributing to the gender disparities in academic authorship, especially in male-dominated fields like urology. Longitudinal studies can assess whether gender concordant mentorship or academic institutional policies are effective in promoting gender equity in the scientific realm.

### Limitations

This study is not without limitations. In this study, gender identification relied on traditional markers (such as names and pronouns), which may not fully capture the diversity of gender identities, we sought to mediate that by using a binary identification method. There were no authors identified on internet search who appeared to be outwardly transgender or non-binary. The analysis was also limited to FA and LA, due to the relative importance of those authorship positions. However, this limits the data collected and notably overlooks contributions of middle authors. Due to this, it is possible that this representation of women authors was missed. Finally, these three journals were chosen based on their prominence in the sexual medicine literature and are meant to be three of the most representative journals. However, limiting data collection to three journals affects the generalizability of the data to the rest of the sexual medicine literature.

It is important to note, the senior author of this paper is male, this, as a byproduct will seem like an oxymoron given the content of this manuscript. However, it is crucial to state, that regardless of identifying as male or female, strong encouragement and allyship is necessary from any senior author to bring forth the changes that we are hoping to achieve in the field of urology with reducing gender disparities in the academic publishing realm.

## Conclusion

Despite the growing representation of women in medicine, even in the predominantly male field of urology, significant strides must be made to achieve gender equity in sexual medicine authorship. This study highlighted the need for increased authorship by women in the field of sexual medicine; we encourage specific opportunities to improve this via institutional support and individual mentorship.
